# A Fuzzy Fusion Rotating Machinery Fault Diagnosis Framework Based on the Enhancement Deep Convolutional Neural Networks

**DOI:** 10.3390/s22020671

**Published:** 2022-01-16

**Authors:** Daoguang Yang, Hamid Reza Karimi, Len Gelman

**Affiliations:** 1 Department of Mechnical Engineering, Politecnico di Milano, 20156 Milan, Italy; daoguang.yang@polimi.it; 2 School of Computing and Engineering, University of Huddersfield, Huddersfield HD1 3DH, UK; L.Gelman@hud.ac.uk

**Keywords:** Convolutional Neural Network, rotating machinery, fuzzy fusion, fault diagnosis

## Abstract

Some artificial intelligence algorithms have gained much attention in the rotating machinery fault diagnosis due to their robust nonlinear regression properties. In addition, existing deep learning algorithms are usually dependent on single signal features, which would lead to the loss of some information or incomplete use of the information in the signal. To address this problem, three kinds of popular signal processing methods, including Fast Fourier Transform (FFT), Short-Time Fourier Transform (STFT) and directly slicing one-dimensional data into the two-dimensional matrix, are used to create four different datasets from raw vibration signal as the input data of four enhancement Convolutional Neural Networks (CNN) models. Then, a fuzzy fusion strategy is used to fuse the output of four CNN models that could analyze the importance of each classifier and explore the interaction index between each classifier, which is different from conventional fusion strategies. To show the performance of the proposed model, an artificial fault bearing dataset and a real-world bearing dataset are used to test the feature extraction capability of the model. The good anti-noise and interpretation characteristics of the proposed method are demonstrated as well.

## 1. Introduction

Rotating machinery is a key element in the modern industry, in particular within the context of the fourth industrial revolution or Industry 4.0. Especially, gears and bearings are essential components of rotating machinery and any faults in gears or bearings can lead to machine failure, which can result in accidents, product unavailability, and financial losses [1]. Hence, it is necessary to develop an efficient method that could identify the fault as early as possible [2]. The traditional method is to perform signal analysis to identify the fault type for rotating machinery, for instance, an acoustic signal, a current signal, or vibration signal are often used for fault prognosis purposes [3]. Vibration signal analysis is the most widely used method to identify some particular faults [4]. Nowadays, most of the fault diagnosis methods are usually based on the vibration signals or the signals transformed from the vibration signals based upon several popular signal processing techniques, including fast Fourier transform (FFT), wavelet transform (WT), and short-time Fourier transform (STFT). Expert fault diagnosis knowledge in the traditional rotating machinery fault diagnosis is usually needed to extract effective features from the input data [5,6].

With the development of deep learning algorithms, multiple layers are used to progressively extract fault features from the input dataset [7,8,9]. Firstly, based on the strong feature extraction and decision-making abilities of the deep learning algorithms, such as Convolutional Neural Networks (CNN) [10,11], Recurrent Neural Networks (RNN) [12], Generative Adversarial Networks (GAN) [13], and deep reinforcement learning [14], they have been increasingly used for the fault diagnosis context in recent years. For example, it has been shown that CNN is specially suitable at dealing with 1-D, 2-D, or 3-D datasets [7,15], RNN excels with time-series data. So RNN is a popular method for prognostic and health management [16], GAN is good to handle datasets with imbalanced samples [13] and deep reinforcement learning could treat rotating machinery fault diagnosis problem as an end-to-end learning process [17]. In literature, it is shown that different deep learning algorithms can process raw vibration signals well when the signal is not complex. In addition, the signals are more easily identified by deep learning models after they have been processed by some signal processing methods because that could effectively reduce the difficulty of the deep learning model to extract features from signals [18].

However, different signal processing methods would extract different features from the raw vibration signal, which would also lead to the loss of some information in the signal. For example, the FFT would transform the time-domain signal into the frequency-domain signal, which would lose the time-domain information [19]. The STFT would save the information regarding the energy distribution of the signal in time and frequency domains, but there is a limit to the amount of time-domain information it extracts [20]. In addition, different types of deep learning models have shown different capabilities for feature extraction in time or frequency domains. For instance, shallow learning algorithms, like multi-layer perceptrons and the support vector machines are usually used to deal with low-dimensional data due to their weak ability to extract features from high-dimensional data [8]. Auto-encoder and RNN have a strong ability to extract efficient features from a frequency-domain signal. CNN has a powerful feature extraction ability from an image [2,8]. In addition, it is difficult to get the amount of fault data to train a deep learning model which could precisely anticipate the fault type in the real world [21]. However, the CNN has many shortcomings, due to the limitation of the traditional pooling layers and activation functions. For example, the traditional pooling layers result in the loss of original information. The Sigmoid and other traditional activation functions have the problem of gradient vanishing. Therefore, SoftPool and Mish activation functions are proposed to deal with these problems [7,8].

Besides, the above-mentioned results are only based on the implementation of single classifiers with one type of signal. The fusion of different classification models with multiple types of signals is still rare [6]. In principle, there are three kinds of fusion strategies, including data-level fusion, feature-level fusion, and decision-level fusion. The performance of data-level fusion and feature-level fusion is highly dependent on the data. And, the decision-level fusion strategy is more practical than the other two fusion strategies [22,23]. To get a higher accuracy based on the existing model and data, developing a decision-level fusion model could improve the fault identification accuracy by combining several deep learning models with different signals [24]. However, the advantages and disadvantages of different deep learning models and fusion strategies are also different. For example, on the one hand, a deep auto-encoder could rapidly extract effective features from low-dimensional signals, but the results are more ordinary for high-dimensional signals [2,25]. CNN have strong feature extraction capability and can be used to process high-dimensional data, but they are prone to overfitting and the risk of gradient vanishing [10,26]. On the other hand, the conventional fusion strategies are vulnerable to poor quality of data, and they usually fail to demonstrate the importance of each classifier and interaction between each classifier. Fuzzy fusion is a good method that could integrate the important information in the poor classifier, which is seldom used to fusion classifiers in the fault diagnosis field. The above-mentioned shortcomings motivate the authors to develop the novel model, that is proposed here [6].

In this article, to solve the aforementioned problems and improve the performance of fault diagnosis, a highly accurate intelligence fault diagnosis framework is developed with four different signals based on an explainable fusion framework. The results demonstrate better performance than other existing methods. The main contributions of this paper are highlighted as follows:

(1) To make full use of the available data, a new intelligence fault diagnosis framework is developed to improve the performance of fault type identification based on four kinds of signals, including the raw vibration signal and other signals transformed from raw vibration signal.

(2) An enhancement CNN structure is developed with Soft Pool layers and Mish activation function, which could take advantage of the information from signals and overcome the training problems in the conventional CNN. The results show that this enhancement CNN structure has better feature extraction capability than the traditional CNN with normal data and noise data.

(3) Fuzzy integral and Shapley scores are developed for classifiers fusion and analysis of the importance of each classifier at the decision-level to address the problems of conventional fusion strategies, such as, the results of traditional fusion strategies are very susceptible to the influence of poorly powerful classifiers and it is difficult to analyze the interaction between classifiers based on the traditional fusion strategies.

The remainder of this paper is organized as follows. Section 2 presents the knowledge of signal processing techniques and the basic theory of CNN. Section 3 illustrates the structure of the proposed model in detail. In Section 4, the performance of the proposed model is evaluated on two rotating machinery fault datasets. Finally, Section 5 concludes this paper.

## 2. Background and Related Work

This section firstly describes the related knowledge, which would be used in subsequent parts of this article, including two popular signal processing approaches, FFT and STFT, the brief introduction of convolutional neural networks (CNN), and two kinds of empirical fusion strategies, including Majority Voting and Average Fusion.

### 2.1. Signal Processing Techniques

Signal processing techniques usually are essential tools in rotating machinery fault diagnosis that could minimize the difficulty of identifying the fault types, not only traditional fault diagnosis but also deep learning-based fault diagnosis and data-driven fault diagnosis. Among these approaches, FFT and STFT are currently widely used in signal processing methods for rotating machinery fault diagnosis.

Fast Fourier transform (FFT) is a function that could transform a time-domain signal into a frequency-domain signal. FFT is based on the discrete Fourier transform (DFT), but the computation speed is faster than DFT due to the lower computation complexity [27]. The DFT function is defined as follows:

(1) $$X_{k}=\sum_{n=0}^{N-1}x_{n}e^{-\frac{i2\pi nk}{N}},\left(k=0,\ldots ,N-1\right)$$

where $X_{k}$ is the DFT of the sequence $x_{n}$.

Due to the characteristics of rotating machinery fault signals, the vibration signal would usually be transformed into a frequency-domain signal for further analysis [28].

STFT is a time-frequency analysis method, unlike FFT, the 1-D vibration signal would be transformed into a 2-D signal by STFT. Hence, STFT is often used to get 2-D data as the input of 2-D convolutional layers, the first layer of CNN. The transformed signal by STFT would display the time and frequency domain information in the signal [29]. The basic STFT calculation equation is expressed as follows:

(2) $$STFT\left\{x\left(t\right)\right\}\left(t,w\right)=\int_{-\infty }^{\infty }x\left(s\right)g\left(s-t\right)e^{-jwt}ds$$

where x(t) is the time-domain signal, *t* denotes the time, *w* denotes the frequency and g(s−t) is a window function whose center is at time *t*.

Although STFT presents time and frequency domain information in the signal, the length of the window function of the STFT formula directly affects the resolution of the time and frequency domain information. The shorter the window function demonstrates higher resolution time-domain information, and conversely shows higher resolution frequency domain information. Hence, a reasonable length of window function can reduce the difficulty of signal analysis based on different signals. For more details, the time resolution computing equation is shown by

(3) $$T=\left[\frac{N_{x}-N_{o}}{N_{w}-N_{o}}\right]$$

where $\left[\cdot \right]$ indicates a round down function. $N_{x}$, $N_{o}$, $N_{w}$ are the length of the signal prepared for processing, the length of the signal overlap during the processing of the window function, and the length of the window function, respectively. Besides, the frequency resolution function is given by

(4) F=NwNw22+1,ifNwisevenNw+1Nw+122,ifNwisodd

### 2.2. General Introduction of Conventional CNN

Conventional CNN usually constitutes several convolutional layers, pooling layers, and a fully connected layer. To avoid overfitting and speed up convergence, dropout layers and normalization layers are introduced into the structure of CNN [26]. 1-dimensional (1-D) and 2-dimensional (2-D) input depth feature maps can be obtained on account of the convolution and pooling layers. The calculation equation of the *l*th convolutional layer is shown as follows:

(5) $$x_{j}^{l}=f\left(\sum_{i\in M_{j}}x_{i}^{l-1}\cdot K_{ij}^{l}+b_{j}^{l}\right)$$

(6) $$x_{i,j}^{k,l}=f\left(\sum_{k=1}^{K}\sum_{i,j=1}^{N_{U}}\sum_{q,p=1}^{N_{U}}U_{q,p}^{k,l}*x_{i+q-1,j+p-1}^{k,l-1}+b^{k,l}\right)$$

where ∗ indicates the convolution operation. *l* indicates the number of the network layer, *K* is the convolutional kernel, *b* is the bias, $x_{j}^{l}$ and $x_{i,j}^{k,l}$ denote the output of the *l*th convolutional layer, $x_{j}^{l-1}$ and $x_{i+q-1,j+p-1}^{k,l-1}$ denotes the input of the *l*th layer, $M_{j}$ indicates the input set of features, and $f\left(\cdot \right)$ is the activation function used in CNN, such as Rectified linear unit (ReLU).

The pooling layer is usually located after the convolution layer to reduce the computational complexity and extract features. The popular pooling layers are max pooling and average pooling layers [30]. The advantages and disadvantages of max pooling and average pooling will demonstrate in the next section. The functions of max pooling and average pooling operation are respectively demonstrated as follows:

(7) $$y=maxp\left(x\right)$$

(8) $$y=avep\left(x\right)$$

where $maxp\left(\cdot \right)$ represents max pooling operation, and $avep\left(\cdot \right)$ represents average pooling operation.

### 2.3. Conventional Fusion Strategies

There are several kinds of traditional fusion methods, including non-trainable methods (Majority voting, Average, etc.). Majority voting and Average methods could be directly used to combine the individual classifier outputs, but they are vulnerable to poor results. Majority voting is a kind of hard voting method, while Average could be seen as a kind of soft-voting method, in which the target label is computed by the average probability.

Majority voting is a simple fusion method, which is usually used in ensemble learning to deal with classification problems. The key idea of Majority voting is to count the number of the predicted classes which was the output of each classifier. If the fusion does not result in an optimal value, one of the results will be selected randomly [31,32].

The computation process of Average fusion is to average the output values of the softmax layer in each classifier. The performance of Average fusion is easily influenced by the worst output values [24].

## 3. Framework of the Proposed Method

**Figure 1:**
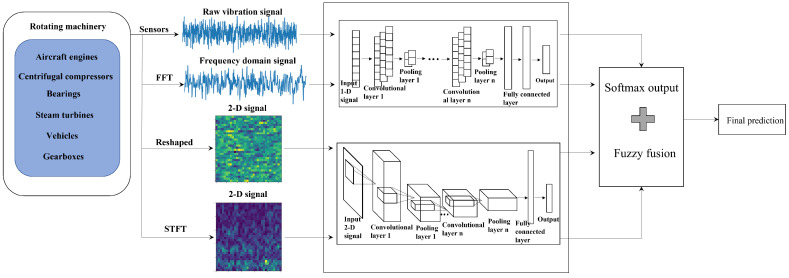
A general architecture of the designed fusion method.

In this section, a CNN-based decision fusion model is proposed for rotating machinery fault diagnosis, which consists of the following steps. Step 1: Collect vibration signals. Step 2: Transform the raw vibration signals into Frequency domain (transformed by FFT) and 2-D signals (directly sliced or transformed by STFT). Step 3: Build CNN models, replace the pooling layers and activation function with soft pooling and Mish activation function. Step 4: Train each CNN by different signals. Step 5: Design a fuzzy decision fusion strategy to fuse all outputs of CNN models. The general architecture of the designed fusion method is demonstrated in Figure 1, and specific details are demonstrated in the subsections below.

### 3.1. Enhancement CNN Models

Although the conventional CNN is used in many fields due to its powerful performance, much attention has been paid to improving its pooling strategy and activation function for improving capabilities. There are some disadvantages of max pooling and average pooling. For instance, max-pooling just utilizes the most powerful feature elements, which is unavoidable to cause the loss of valuable data in the signals used for fault diagnosis. Average pooling loses particular attention to the contribution of feature elements because different feature elements are always considered equally. Besides, the certainty of the max pooling and average pooling will additionally raise the possibility of over-fitting.

Soft pooling(SoftPool) is a strategy recently introduced to address the restrictions of max pooling and average pooling [33]. The mathematical equation of SoftPool is given as follows:

(9) $$w_{i}=\frac{e^{a_{i}}}{\sum_{j\in R}e^{a_{j}}}$$

(10) $$\tilde{a}=\sum_{i\in R}w_{i}\cdot a_{i}$$

where *R* is the kernel region, $a_{i}$ is the feature value within region *R*, *i* is the index in region *R*, $w_{i}$ is the weight transformed from feature value. $\tilde{a}$ is the output value of Soft pooling.

SoftPool is a more balanced method than max pooling or average pooling which simply selects the average value or maximum value. In this case, it would preserve the maximum feature value, which would ensure that the most important feature is maintained. On the other hand, it also does not minimize the strength of the features within the calculation region as the average pooling does.

The activation function is a key component of the neural network structure, which would avoid high calculation cost, overfitting, and gradient vanishing. For example, Sigmoid and Tanh functions have these drawbacks. Although ReLU was developed to deal with these problems, it would stop upgrading parameters when the inputs are all negative. Hence, it is necessary to develop a better activation function to get better performance. Here, the Mish activation function is proposed [34], which is similar to the Swish function. Mish activation function is defined as

(11) $$f\left(x\right)=x\tanh\left(softplus\left(x\right)\right)$$

where $softplus\left(x\right)=x\tanh\left(\ln\left(1+e^{x}\right)\right)$.

### 3.2. Decision-Level Fuzzy Fusion Strategy

**Figure 2:**
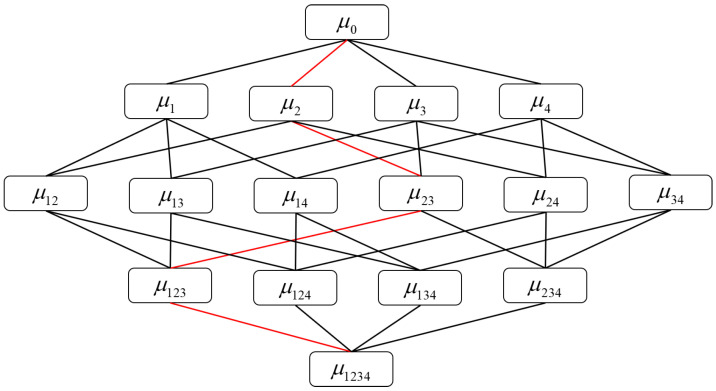
Lattice visualization for four classifiers based on fuzzy measures.

After training the modified CNN model with different signals, to take full advantage of the vibration signals, it is necessary to develop an explainable ensemble model. Average value fusion and majority voting have been the most popularly adopted fusion methods for ensemble learning models. Nevertheless, the outputs provided by submodels are always treated equally in these two fusion strategies, which would enhance the contributions of worse outputs and do not explain the interaction between each classifier. Based on the benefits of the fuzzy integral [35], such as the explainable characteristics, it could explain not only the importance of each classifier but also the interaction between classifiers. For example, firstly, as shown in Figure 2, the red line could demonstrate the calculation order of fuzzy integral. Secondly, the Shapley index and interaction index are used to analysis the importance of each classifier and the interaction between classifiers, which could enhance the explainable ability of the fusion strategy. The Shapley index is used to demonstrate the contribution of the sources. And the interaction index could explain how well classifiers interact with each other [36].

Hence, an improved explainable decision-level fusion strategy based on the fuzzy measure and fuzzy integral is proposed in the sequel.

Step 1: According to the accuracy of each CNN model, set fuzzy measures *G*. According to the SoftMax layer of each CNN model based on different features, set confidence scores *S*. The fuzzy measure g(e) has the following monotonic property:

(12) $$e_{i}\subset e_{j}\Rightarrow g\left(e_{i}\right)\leq g\left(e_{j}\right)$$

Based on the Sugeno fuzzy-λ measures, if $e_{i}\cap e_{j}=\emptyset$, there exists λ>−1, such that

(13) $$g\left(e_{i}\cup e_{j}\right)=g\left(e_{i}\right)+g\left(e_{j}\right)+\lambda g\left(e_{i}\right)g\left(e_{j}\right)$$

With the attributes developed, λ can likely be acquired from this equation.

(14) $$\lambda +1=\prod_{n=1}^{N}\left(\lambda g\left(e_{n}\right)+1\right)$$

where *N* is the number of values in confidence scores *S*, here the features in our cases are 4. So λ is obtained from this equation which is greater than −1.

Step 2: Based on the Choquet integral, it allows for all linear ensembles as well as a wider range of combinations of empirical fusion strategies, such as average and majority voting. The fuzzy fusion can be operated by this formula:

(15) $$C_{g}\left(S\right)=\sum_{n=1}^{N}S_{\pi n}\left[g\left(e_{\pi i}\right)-g\left(e_{\pi i-1}\right)\right]$$

where confidence scores *S* is replaced to $S_{\pi }$, that is

(16) $$S_{\pi 1}\geq S_{\pi 2}\geq ,\ldots ,\geq S_{\pi N-1}\geq S_{\pi N}$$

and $e_{\pi i}$ is the subset of the *i* maximum scores in $S_{\pi }$ acquired by applying Sugeno fuzzy-λ approaches.

Step 3: Using Shapley index and Interactive index to analyze the classifier ranking and the interaction between each classifier.

The formula of the Shapley index is given as follows:

(17) $$\left\{\begin{array}{c} \Phi_{\mu }\left(i\right)=\sum_{K\subseteq X\backslash \left\{i\right\}}\zeta_{X,1}\left(K\right)\left(\mu \left(K\cup \left\{i\right\}\right)-\mu \left(K\right)\right)\\ \zeta_{X,1}\left(K\right)=\frac{\left(\left|X\right|-\left|K\right|-1\right)!\left|K\right|!}{\left|X\right|!} \end{array}\right.$$

where $X\backslash \left\{i\right\}$ means the subset *X* (all classifiers) does not include classifier *i*. The Shapley value of μ is the vector $\Phi_{\mu }=\left[\Phi_{\mu }\left(1\right),\cdots ,\Phi_{\mu }\left(N\right)\right]$, where $\sum_{i=1}^{N}\Phi_{\mu }\left(i\right)=1$. The Shapley index can be explained as the average contribution value of each classifier *i* among all classifiers.

The equation of the interaction index is expressed as follows:

(18) $$\left\{\begin{array}{c} I_{\mu }\left(i,j\right)=\sum_{K\subseteq X\backslash \left\{i,j\right\}}\zeta_{X,2}\left(K\right)\left(\mu \left(K\cup \left\{i,j\right\}\right)-\mu \left(K\cup \left\{i\right\}\right)-\mu \left(K\cup \left\{j\right\}\right)+\mu \left(K\right)\right)\\ \zeta_{X,2}\left(K\right)=\frac{\left(\left|X\right|-\left|K\right|-2\right)!\left|K\right|!}{\left(\left|X\right|-1\right)!} \end{array}\right.$$

**Figure 3:**
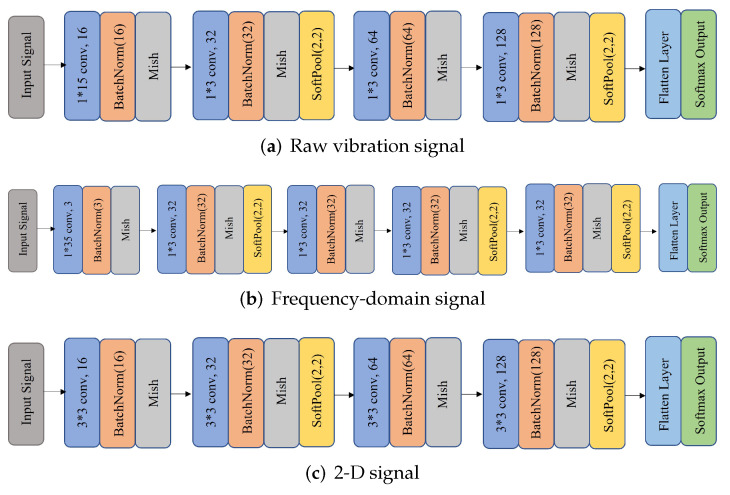
CNN structures used in the proposed method. (**a**) the structure based on the raw vibration signal, (**b**) the structure based on the frequency-domain signal, (**c**) the structure based on the 2-dimensional data, including the raw vibration signal directly reshaped into 2-D signal and transformed by STFT.

The structures of the CNN classifiers are shown in Figure 3. The CNNs used as classifiers consist of the convolutional layer, batch normalization layer, and pooling layer. The raw vibration signal and frequency-domain signal are subject to a wide kernel in the first convolutional layer. The activation function in these four CNN is the Mish function. And, the pooling layer is Soft Pooling.

## 4. Experiments

### 4.1. Datasets

There are several open-source rotating machinery fault diagnoses, such as the bearing fault dataset of Case Western Reserve University (CWRU), the bearing fault dataset of Society for Machinery Failure Prevention Technology (MFPT), and the bearing fault dataset of Paderborn University (PU), etc. Due to the easy identification of samples in the CWRU bearing datasets compared to other databases, this section focuses on the performance of the proposed model in the MFPT bearing dataset and PU bearing dataset.

**Figure 4:**
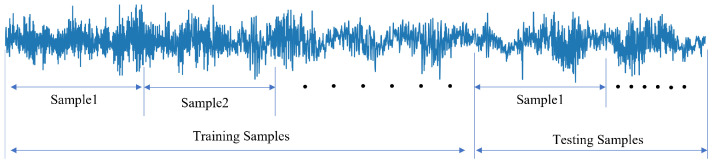
The data processing of raw vibration signals.

**Figure 5:**
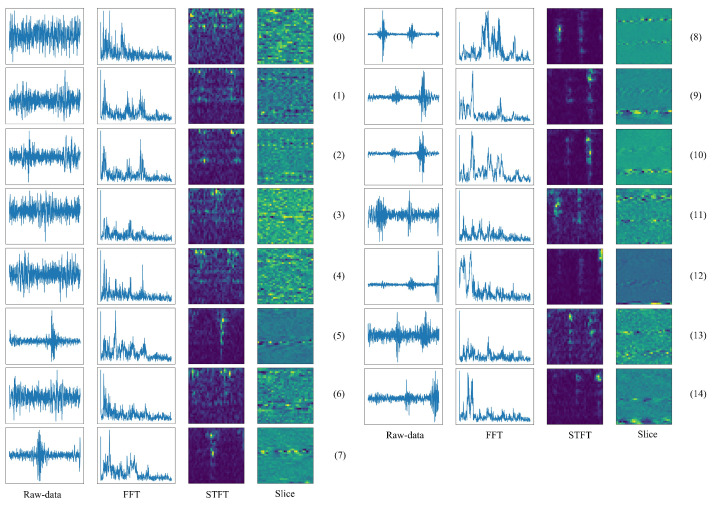
Four kinds of different input signals from the MFPT bearing dataset.

MFPT bearing dataset (Artificial fault bearing dataset) contains three main health conditions: normal state, inner race fault state, and outer race fault state [37]. The normal state data was gathered under 270 lbs load, the outer race fault state data were gathered under seven different loads, respectively, 25, 50, 100, 150, 200, 250, and 300 lbs of load, and the inner race fault state data were also gathered under seven different loads, respectively, 0, 50, 100, 150, 200, 250, and 300 lbs of load. The same fault type would contain different information under different loads. Hence, there are seven labels in the inner race fault state and outer race fault state. There is a total of 15 labels in this case. In addition, to test the performance of the proposed model, the training sample does not contain any replicated information, and the testing samples don’t contain any information from training samples. Here, each sample of the raw vibration signals contains 1024 points and is then transformed into other kinds of signals, including frequency-domain signal, time-frequency domain signal and two-dimensional signal. The data processing of the raw vibration signals is shown in Figure 4. Visualization of the four different input signals based on the MFPT bearing dataset is illustrated in Figure 5. In each class of the MFPT bearing dataset, there are 120 samples to train and 30 samples to test the performance of the model.

**Figure 6:**
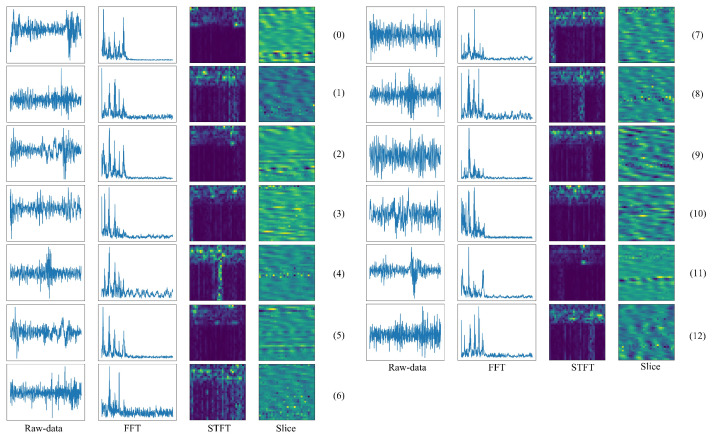
Four kinds of different input signals from the PU-bearing dataset.

To show the performance of the proposed model in the real-world dataset, Paderborn University (PU) bearing dataset contains 13 kinds of real damages caused by accelerated lifetime tests [38]. Here, the details of the used fault datasets are described as follows: the bearing rotating speed is 1500 rpm; the load is 0.7 Nm; the radial force is 1000 N. Hence, there is a 13-class classification task in each load. The detail of the training samples is the same as the MFPT bearing dataset, which is based on the size of the datasets. Visualization of four different input signals based on PU bearing dataset is illustrated in Figure 6. In each class of the PU bearing dataset, there are 200 samples to train and 50 samples to test the performance of the model.

### 4.2. Implementation

The input dimensions for raw vibration signals and FFT signals are 1024, 512 and the size of input images for the slice signal and STFT signal is 32 × 32, 33 × 33 on MFPT bearing dataset and PU bearing dataset. The sample sizes for each operation state in the MFPT bearing dataset and PU bearing dataset were 120 and 200, respectively. The CNN classifiers are trained with Adaptive Moment Estimation (Adam) with an initial learning rate of 0.001. The models are trained for up to 200 epochs, with a batch size of 100, 200, respectively, in the MFPT bearing dataset and PU bearing dataset. No form of data augmentation is used in our method.

In addition, the proposed model is programmed with Python 3.7.12 with Pytorch 1.9.0 (Machine learning framework) and executed on the Windows 10 operating system based on Google Colaboratory.

### 4.3. Training Process Analysis

**Figure 7:**
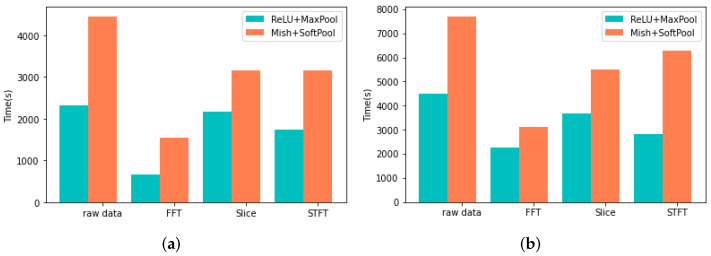
Training time of the modified CNN and conventional CNN. (**a**) MFPT bearing dataset. (**b**) PU bearing dataset.

**Figure 8:**
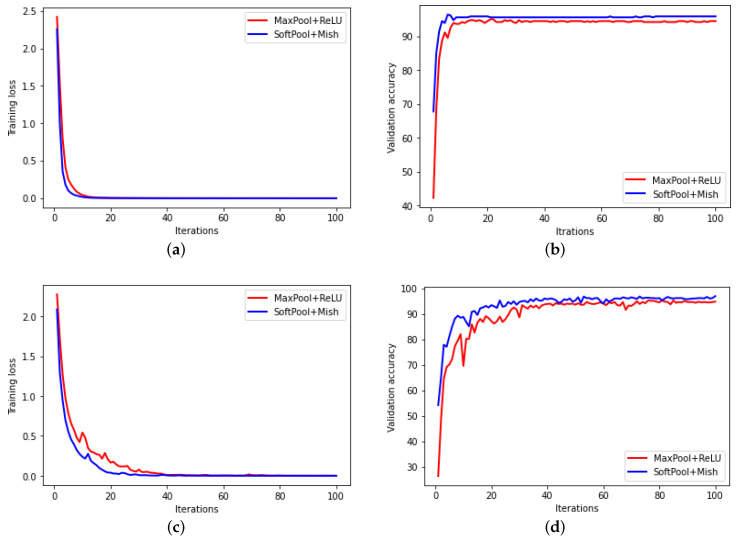
Training process of the modified CNN and conventional CNN. (**a**) MFPT training loss. (**b**) MFPT validation accuracy. (**c**) PU training loss. (**d**) PU validation accuracy.

To display the advantages and disadvantages of the proposed method, it is necessary to compare the time spent on the modified CNN model with the traditional CNN model. The time spent on these two kinds of CNN models that were trained with MFPT bearing dataset and PU bearing dataset was shown in Figure 7. In this case, the training epochs of models were 200. As there is no GPU speed up the calculation, the time cost is more than 10 min. In addition, according to the basic theory of SoftPool and Mish activation functions, as mentioned above, the computation complexities of SoftPool and Mish activation functions are higher than MaxPool and ReLU, so the time cost of the proposed model is two times bigger than the traditional CNN. Even if the training time of the enhancement CNN model is a little larger than the conventional CNN model, its training process is better than the conventional CNN model (frequency-domain signal), which is shown in Figure 8. The enhancement CNN model could extract more important information than the conventional CNN model.

### 4.4. Comparison of Fuzzy Fusion with Empirical Fusion

The choice of fuzzy measures is initialized based upon the performance of the individual classifiers as tabulated in Table 1. In addition, as shown in Table 1, the CNN model using Mish activation function and SoftPool would perform better than the CNN model using ReLU activation function and MaxPool. As shown in Table 2 and Table 3, the proposed CNN model was tested with noise data, in this case, the proposed method also has a better performance than the traditional CNN. We take the average accuracy as the initial fuzzy measure for every modified classifier. The fuzzy measures are then experimentally refined through a search in their neighborhood, which yields 0.05, 0.2, 0.01, 0.07 as the choice of fuzzy measures which is the best performance for MFPT bearing dataset. To show the performance of the fusion strategy, the PU-bearing database does not use the fusion algorithm here with the normal data. Here, the noise data of PU bearing data (SNR = 4) was used to test the performance of the proposed fusion method.

To test the performance of the proposed fusion method, it is compared with two empirical fusion strategies, including average and majority vote. Table 4 shows the results of our experiments. Fuzzy fusion could effectively utilize each classifier based on their own performance on different datasets, which is different from the Average fusion strategy and Majority Vote strategy. As shown in Table 4, the Average strategy and Majority Vote strategy are easy to be affected by the worst classifier, which could even lead to worse performance.

### 4.5. Classifier Ranking and Interactive

**Figure 9:**
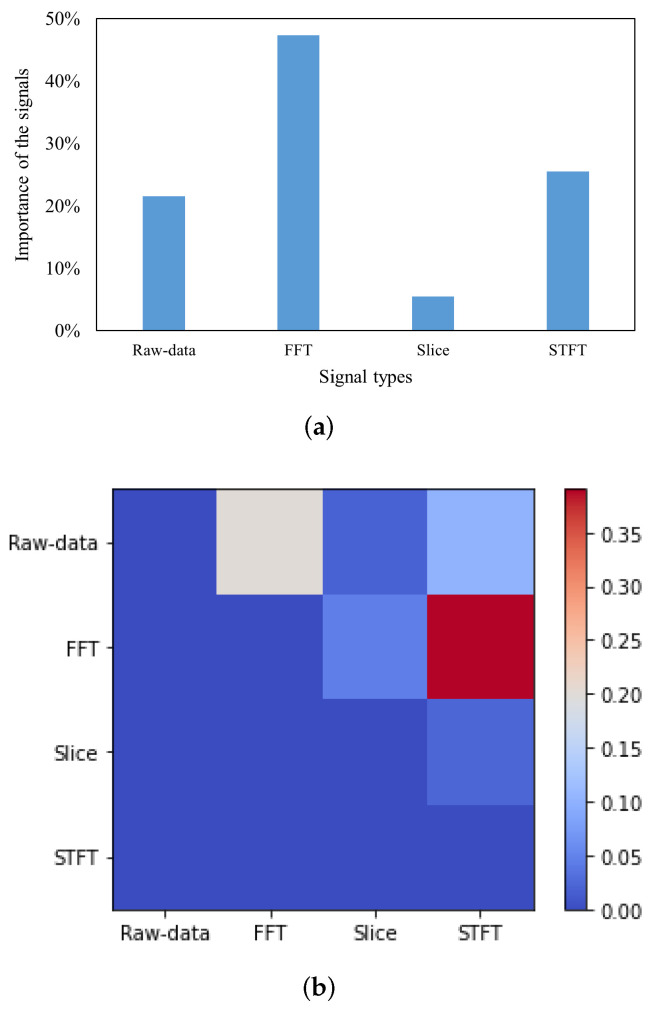
The explainable features of fuzzy fusion with MFPT bearing fault dataset. (**a**) The contributions of each signal in fuzzy fusion. (**b**) The interaction index among signals.

Fuzzy fusion is different from the empirical fusion strategy. It can be used to analyze not only the contribution of individual signals in the fusion process but also the influence of signals on each other, which is based on the Shapley index and Interactive index that are strictly computed from the fuzzy measure. For the MFPT bearing dataset without any noise, as shown in Figure 9a, the FFT signal plays the most important role in the fusion process (close to 50% contribution) followed by the STFT signal. In addition, the interaction index among four kinds of signals is shown in Figure 9b. It can be seen that there is positive interaction between signals, and there is the largest positive interactive value between the FFT signal and STFT signal.

**Figure 10:**
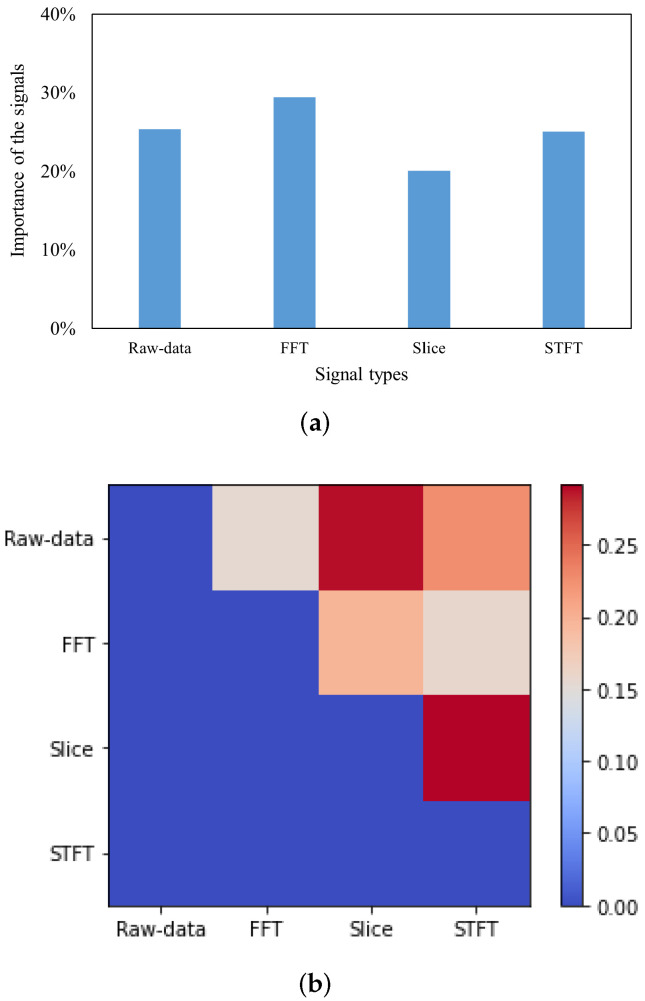
The explainable features of fuzzy fusion with PU bearing fault dataset. (**a**) The contributions of each signal in fuzzy fusion. (**b**) The interaction index among signals.

For the PU bearing dataset that added some noise, which could demonstrate the capability of the proposed model in the real-world environment, the analysis is shown in Figure 10. As signals of non-artificial faults in the PU bearing dataset are used here, there is a small difference in the performance of the CNN for the four signals. As shown in Figure 10a, based on the Shapley scores, the importance of each classifier could be easily described. The importance of each classifier is similar to the fuzzy measures of each classifier. The classifier based on frequency-domain signals is the most important followed by the classifiers (close to 30% contribution) which the input signals are raw vibration signals or time-frequency domain signals. As shown in Figure 10b, although the classifier based on the Slice signals plays a weaker role in the fusion process than other classifiers, this classifier has a large positive interaction value with the classifier based on raw-data signal and STFT signal, respectively, which are different from MFPT bearing dataset.

In addition, based on the Shapley scores and interactive index, each classifier could make a positive influence on the fusion process in this fault diagnosis problem, unlike the Average Fusion and Majority Vote, which could be influenced by the poor classifier. However, there is a drawback in fuzzy fusion, which is the performance of fuzzy fusion is dependent on the selection of the fuzzy measure. Hence, fuzzy fusion is also a trainable fusion strategy that could be conveniently used to fusion other information sources.

### 4.6. Comparison with State-of-the-Art

MFPT: Table 5 shows a comparison with previous methods. Many deep learning methods based on MFPT bearing datasets usually considered three health states, which would simplify the identification task of these models. These models usually have the best performance to the FFT signal than other kinds of signals, except for the CNN used in [2]. The structures of the models(BiLSTM, LeNet, AlexNet, and ResNet18) are described in [2]. The structure of a novel CNN is shown in [39]. In addition, shallow machine learning methods (SVM and MLP) are also used to compare the performance with the proposed model [39,40]. The proposed method outperforms the ResNet18, even if only the FFT signal is used. The performance of the proposed framework based on four classifiers is around 3% improvement compared to the classifier with one type of signal.

PU bearing dataset: Table 6 shows a comparison with previous methods based on PU bearing dataset. The proposed method outperforms several kinds of existing state-of-art deep learning models, including BiLSTM, SAE, LeNet, AlexNet [2], which the data processing methods are the same as this paper. The structure of the novel CNN, Direct Training Net(DTN), and Feature Transfer Net(FTN) are shown in [40,42,43], respectively. The structures of the models (BiLSTM, SAE, LeNet, AlexNet, and Resnet18) are described in [2]. The performance of the proposed CNN model based on the frequency domain is the same as ResNet18, which has the powerful ability to deal with classification problems. And, the proposed fusion method could effectively improve the performance of the modified CNN model with noise datasets, which could increase the confidence of the model in predicting complex signals.

## 5. Conclusions

With the development of the modern industry, the consequences of mechanical breakdowns are becoming increasingly unacceptable. Therefore, it is necessary to develop a highly accurate model for fault diagnosis. However, the accuracy of a model for fault identification depends on how many effective identification features can be extracted. Hence, to make full use of the information of the existing signals, this study developed a novel fuzzy fusion method for rotating machinery fault diagnosis. In this method, firstly, SoftPool and Mish activation functions were introduced into traditional convolutional neural networks to enhance the feature extraction ability of CNNs. Secondly, raw vibration signals were transformed into three kinds of signals which were used to train a series of CNNs to supply the outcomes. Thirdly, an explainable fuzzy fusion strategy was introduced into rotating machinery fault diagnosis to improve diagnostic performance and the Shapley index was used to explain the classifier contributions and their interactions index among these classifiers.

As the results demonstrate, the modified CNN had a better performance than conventional CNN even in the noise environment, although they needed more time than the conventional CNN. However, the proposed model could have a more explainable property than empirical fusion strategies, it would provide better performance than a single signal or single model, which could provide more precise and confident diagnosis outcomes. In the future, we will focus on the explainability of the CNN, and examine which features are learned by deep learning concepts.

## Figures and Tables

**Table 1:** Performance of individual CNN classifiers on two benchmark datasets.

Feature Used	MFPT				PU			
	ReLU MaxPool	Mish MaxPool	ReLU SoftPool	Mish SoftPool	ReLU MaxPool	Mish MaxPool	ReLU SoftPool	Mish SoftPool
Raw vibration	84.17%	86.11%	84.44%	88.33%	90.92%	90.98%	92.18%	93.32%
FFT	94.44%	95.56%	96.11%	97.50%	96.09%	96.70%	96.69%	98.62%
Slice	71.19%	72.22%	73.22%	74.44%	92.97%	93.31%	92.34%	93.49%
STFT	86.16%	89.72%	88.56%	90.00%	95.24%	95.27%	95.49%	96.69%

**Table 2:** Comparison with traditional CNN on MFPT gearbox dataset with different SNR values.

Model	Signal	SNR						Average
		0	2	4	6	8	10	
ReLU + MaxPool	Raw	68.05%	71.94%	73.33%	80.55%	81.67%	84.72%	76.71%
	FFT	82.50%	85.83%	89.44%	89.44%	90.28%	91.94%	88.23%
	Slice	41.11%	48.61%	52.50%	55.83%	61.39%	65.00%	54.07%
	STFT	64.44%	68.33%	69.17%	80.55%	81.11%	82.22%	74.30%
Mish + SoftPool	Raw	74.17%	78.89%	80.27%	84.72%	85.55%	87.78%	81.89%
	FFT	85.83%	87.78%	91.11%	92.22%	93.33%	94.17%	90.74%
	Slice	50.28%	52.22%	59.44%	63.33%	64.17%	66.11%	59.26%
	STFT	65.56%	76.94%	78.11%	81.94%	82.78%	84.44%	78.26%

**Table 3:** Comparison with traditional CNNs on PU bearing dataset with different SNR values.

Model	Signal	SNR						Average
		0	2	4	6	8	10	
ReLU + MaxPool	Raw	79.49%	81.95%	85.66%	87.02%	88.08%	88.91%	85.19%
	FFT	81.92%	87.38%	88.91%	92.60%	92.86%	93.12%	89.47%
	Slice	78.54%	83.41%	84.66%	89.03%	89.37%	90.82%	85.97%
	STFT	81.33%	86.66%	87.34%	90.26%	91.03%	91.16%	87.96%
Mish + SoftPool	Raw	83.01%	84.63%	86.42%	89.22%	90.43%	91.79%	87.58%
	FFT	82.48%	89.08%	92.01%	92.58%	94.34%	95.21%	90.95%
	Slice	81.23%	86.18%	88.98%	91.54%	91.34%	92.21%	88.58%
	STFT	85.43%	87.31%	89.52%	90.08%	94.18%	95.79%	90.39%

**Table 4:** Comparison of two empirical fusion strategies with the proposed fusion strategy.

Fusion Strategy	Datasets	
	MFPT	PU
Average	95.55%	96.04%
Majority Vote	96.67%	96.31%
Proposed Fusion	98.06%	96.62%

**Table 5:** Comparison with existing approaches on MFPT bearing dataset.

Method	Year	Feature	Accuracy (%)
BiLSTM [2]	2020	FFT	94.76
LeNet [2]	2020	FFT	94.76
AlexNet [2]	2020	FFT	93.4
ResNet18 [2]	2020	FFT	95.92
SVM [41]	2021	CWT	91.2
MLP [39]	2020	Scalogram	94.00
SVM [39]	2020	Scalogram	92.7
CNN [39]	2020	S-Transform	95.59
CNN [40]	2021	raw-data	79.63
**Proposed**	-	**Integration**	**98.06**

**Table 6:** Comparison with existing approaches on PU bearing dataset.

Method	Year	Feature	Accuracy (%)
BiLSTM [2]	2020	FFT	94.29
SAE [2]	2020	FFT	92.86
CNN [42]	2019	raw-data	95.57
CNN [40]	2021	raw-data	99
DTN [43]	2021	raw-data	95.26
FTN [43]	2021	raw-data	97.79
LeNet [2]	2020	FFT	96.13
AlexNet [2]	2020	FFT	95.57
ResNet18 [2]	2020	FFT	99.48
**Proposed**	-	**Integration**	**99.62**

## Data Availability

The data presented in this study are available from the Internet (Named MFPT gearbox dataset and PU bearing dataset).
